# Inhibition of Hepatitis B Virus by AAV8-Derived CRISPR/SaCas9 Expressed From Liver-Specific Promoters

**DOI:** 10.3389/fmicb.2021.665184

**Published:** 2021-06-26

**Authors:** Kun Yan, Jiangpeng Feng, Xing Liu, Hongyun Wang, Qiaohong Li, Jiali Li, Tianmo Xu, Muhammad Sajid, Hafiz Ullah, Li Zhou, Limin Zhou, Yu Chen

**Affiliations:** ^1^State Key Laboratory of Virology, Modern Virology Research Center, College of Life Sciences, Wuhan University, Wuhan, China; ^2^Animal Biosafety Level III Laboratory at Center for Animal Experiment, School of Basic Medical Sciences, Wuhan University, Wuhan, China; ^3^Department of Gynecology, Maternal and Child Health Hospital of Hubei Province, Tongji Medical College, Huazhong University of Science and Technology, Wuhan, China

**Keywords:** hepatitis B virus, CRISPR/SaCas9, liver-specific promoter, adeno-associated virus, gene therapy

## Abstract

Curative therapies for chronic hepatitis B virus (HBV) infection remain a distant goal, and the persistence of stable covalently closed circular DNA (cccDNA) during HBV replication is a key barrier that is hard to break through using the drugs currently approved for HBV treatment. Due to the accuracy, efficiency, and cost-effectiveness of genome editing, CRISPR/Cas technologies are being widely used for gene therapy and in antiviral strategies. Although CRISPR/Cas could possibly clear cccDNA, ensuring its safety is requirement for application. In our study, we analyzed the liver specificity of several promoters and constructed candidate promoters in the CRISPR/*Staphylococcus aureus* Cas9 (SaCas9) system combined with hepatotropic AAV8 (whereby AAV refers to adeno-associated virus) to verify the efficacy against HBV. The results revealed that the reconstructed CRISPR/SaCas9 system in which the original promoter replaced with a liver-specific promoter could still inhibit HBV replication both *in vitro* and *in vivo*. Three functional guide RNAs (gRNAs), T_2_, T_3_, and T_6_, which target the conserved regions of different HBV genotypes, demonstrated consistently better anti-HBV effects with different liver-specific promoters. Moreover, the three gRNAs inhibited the replication of HBV genotypes A, B, and C to varying degrees. Under the action of the EnhII-Pa1AT promoter and AAV8, the expression of SaCas9 was further decreased in other organs or tissues in comparison to liver. These results are helpful for clinical applications in liver by ensuring the effects of the CRISPR/Cas9 system remain restricted to liver and, thereby, reducing the probability of undesired and harmful effects through nonspecific targeting in other organs.

## Introduction

Hepatitis B virus (HBV) infection remains a major public health burden. One-third of people worldwide have been exposed to HBV, of which about 257 million are chronically infected according to a WHO report (Revill et al., 2019). Although effective preventive vaccines developed for HBV have been in use for decades, there is no effective treatment. Viral hepatitis is still one of the top 10 causes of death in the world today due to the large population of infected people, and HBV accounted for near half of viral hepatitis-related mortality (Stanaway et al., 2016).

At present, the approved HBV treatment drugs mainly include reverse transcriptase inhibitors and immune mediating factors. Among the reverse transcriptase inhibitors are nucleoside analogs (NUCs), including lamivudine, adefovir dipivoxil, telbivudine, entecavir, and tenofovir (Terrault et al., 2016; Liver, 2017). NUCs can effectively inhibit the reverse transcription of HBV by directly acting on HBV reverse transcriptase, reducing the virus to a level below the detection limit; however, due to the nuclear closure of NUCs, they are ineffective against covalently closed circular DNA (cccDNA; Zoulim et al., 2016).

After treatment ceases, HBV can continue to use cccDNA as a template to produce progeny viruses, and viremia recurs. Of concern is that long-term use may result in the development of drug-resistant mutation strains. Interferon (IFN), which is an immune-mediated factor, achieves antiviral effects by regulating innate and acquired immune responses, which could clear infected cells to achieve a functional cure for HBV infection. However, the rate of treatment success is low and often accompanied by side effects (Woo et al., 2017). Virus replication may return to normal levels at any time after drug withdrawal or drug resistance occurs (Zoulim and Locarnini, 2009; Deng and Tang, 2011). cccDNA serves as a template for the transcription of viral RNAs through employing the cellular transcription machinery (Rall et al., 1983; Shi and Zheng, 2020), which is resistant to common antiviral therapies. The key challenge is the persistence of cccDNA in infected hepatocytes. Therefore, specific treatments targeting cccDNA have become an important direction of the current research.

Given its ability to suppress viruses along with the current lack of an effective cure, the CRISPR/Cas9 system has been used as part of a potential therapeutic method in which conserved HBV DNA sequences are targeted to inhibit HBV replication (Abe et al., 2014; Lin et al., 2014; Dong et al., 2015). Although data acquired from experimental models look promising, challenges that are broadly associated with genetic editing therapies need to be met for the approach to be successful against chronic HBV infection. For future clinical applications, one of the most critical issues is safety. Since HBV is a hepatotropic virus, restricting the effects of the CRISPR/Cas9 system to liver is a promising strategy for improving safety.

Adeno-associated virus (AAV) emerged as an ideal delivery tool due to its high viral titer capability with the potential for transduction of all virus-infected cells within a patient. The targeted delivery of AAV to a certain tissue could be achieved by recombinant engineering of an AAV capsid protein with a tissue tropism for an intended infection site. The use of recombinant adeno-associated virus as gene carrier proved to be helpful in gene therapy, provided a safe and effective delivery approach and prompting a series of related studies (Scott et al., 2017; Wang et al., 2017; Liu et al., 2018). In addition, having an established record of safety and its lack of integration properties makes AAV appear to be an ideal candidate for the delivery of CRISPR/Cas9 (Balakrishnan and Jayandharan, 2014). Consequently, several groups have already demonstrated the feasibility of such an AAV delivery method in CRISPR/Cas9-based antiviral studies (Chen et al., 2018). *Staphylococcus aureus* (Sa) Cas9 is approximately 25% smaller than *Streptococcus pyogenes* (Sp) Cas9 (Kotterman and Schaffer, 2014; Ran et al., 2015); this small size makes it possible to deliver SaCas9 using AAV vectors. Among the various serotypes of AAV, AAV8 and AAV9 have commonly been used for delivery into liver, with high tissue tropism.

In addition, two kinds of liver-specific promoters have been studied, including promoters derived from both the HBV and its host. Previous studies have shown that regulatory elements of the HBV are strong and liver-specific *in vitro* and, therefore, might be useful in hepatic gene therapy (Sandig et al., 1996). In addition, the HBV core promoter linked to EnI and EnII (EII-EI-Pc) and X promoter linked to EnI and EnII (EI-EII-Px) could direct a constant and high-level gene expression *in vivo* (Zhao et al., 2010). Certain liver-specific promoters from hosts were characterized and applied to transcriptional targeting both *in vitro* and *in vivo*, including the mouse albumin (Alb) promoter (Gorski et al., 1986), human α-1 antitrypsin (hAAT) promoter (Hafenrichter et al., 1994a; Kramer et al., 2003), and phosphoenolpyruvate carboxykinase (PEPCK) gene promoter (McGrane et al., 1990; Valera et al., 1994). However, there is still a lack of reports on the effectiveness of AAV8-derived CRISPR/SaCas9 with a liver-specific promoter.

Here, in addition to the application of AAV8 for liver-specific delivery, we used liver-specific promoters to solely induce expression of the SaCas9 protein in hepatic cells. We verified the anti-HBV effects of CRISPR/SaCas9 expressed under liver-specific promoters. We found that T_2_, T_3_, and T_6_, targeting the conserved regions of different HBV genotypes, could inhibit HBV replication steadily and efficiently, in contrast to other tested gRNAs. We selected the promoters EnhII-PEPCK and EnhII-Pa1AT for expression of these three gRNA sequences and further verified their anti-HBV effects.

In this study, we provide evidence that the reconstructed CRISPR/SaCas9 system, whose cytomegalovirus (CMV) promoter was replaced with a liver-specific promoter, could still profoundly inhibit HBV replication both *in vitro* and *in vivo*. Both the use of liver-specific promoters and the choice of AAV8 virus delivery vector could improve hepatic specificity. In conclusion, the AAV8-derived CRISPR/SaCas9 system with liver-specific promoters demonstrated prominent anti-HBV effects and liver-specific expression of the transduced genes in mouse.

## Materials and Methods

### Plasmids

The human codon-optimized SaCas9 and chimeric gRNA expression plasmid pX601 were obtained from Addgene (plasmid 61591). The reproduced rcccDNA system, including plasmid prcccDNA-shB2M (genotype D: GenBank accession no. V01460.1) and pCMV-KRAB-Cre, was a generous gift from Qiang Deng (Fudan University; Li et al., 2018a). pAAV/HBV1.2 (genotype A: GenBank accession no. AF305422.1) was a generous gift from Pei-jer Chen (National Taiwan University). The HBV replicons (genotype B: GenBank accession no. EU570069.1; genotype C: GenBank accession no. FJ899793.1) were generous gifts from Ying Zhu (Wuhan University). Several candidate promoters were inserted into the SacI and HindIII restriction sites of pGL3-Basic (Promega). Three promoters with linked luciferase fragments were inserted between the XhoI and BamHI restriction sites of pHAGE (Addgene). The pSV-β-gal and pRL-TK plasmids were obtained from Promega.

### Cell Cultures and Transfection

The cells were maintained in Dulbecco’s modified Eagle’s medium (DMEM) supplemented with 10% fetal bovine serum (FBS) at 37°C and 5% CO_2_. All cells were transfected using Neofect (Neofect Biotech) according to the manufacturer’s instructions. The reconstructed pX601 plasmid/HBV-expressing plasmid/pSV-β-gal plasmid ratio was 8:1:1.

### Animal Experiments

For analysis of the activity of three candidate promoters in different tissues, C57BL/6 mice (5 weeks old) were used and separated into three groups (five mice each). The concentrated lentivirus supernatants were injected into the tail vein of the mice. A week later, the mice were sacrificed, and the liver, lung, kidney, spleen, and heart were extracted and homogenized in 1 ml TRIzol reagent (Life Technologies), and total RNA was isolated following the manufacturer’s instructions.

For analysis of the *in vivo* inhibition of HBV by the liver-specific and HBV-targeting SaCas9 system, C57BL/6 mice (4 weeks old) were used and separated into four groups (five mice each). We injected 4 μg prcccDNA-shB2M and 4 μg pCMV-KRAB-Cre into the tail vein of mice within 8–10 s in a volume of saline equivalent to 10% of the mouse body weight. After 7 days, AAV8 containing EnhII-Pa1AT-T_2_, EnhII-Pa1AT-T_6_, EnhII-Pa1AT-T_mix_ (EnhII-Pa1AT-T_2_:EnhII-Pa1AT-T_6_=1:1), or AAV8 containing GFP were intravenously delivered into the mice *via* tail vein injection (200 μl, 2 × 10^11^ vg). Mice were sacrificed 7 days after two injections. Sera were taken for the analysis of HBsAg, HBeAg, and HBV DNA. For HBsAg and HBeAg detection, mice sera were diluted 10 times with DMEM. For HBV RNA analysis, a piece of liver tissue was homogenized for extracting total RNA. HBV core antigen expression in mice livers was analyzed using immunohistochemical staining as described previously (Liu et al., 2015).

All mice were housed in a pathogen-free mouse colony, and the animal experiments were performed according to the 1998 Guide for the Care and Use of Medical Laboratory Animals (Ministry of Health, China). The protocol was approved by the institutional animal care and use committee of Wuhan University (project license WDSKY0201802).

### Dual-Luciferase Assay

The human hepatoma cell lines Huh7, HepG2, stable expression of sodium taurocholate co-transporting polypeptide in the HepG2-derived cells (NTCP), and the non-hepatocellular carcinoma cell lines HeLa and HEK293T were seeded in 24-well dishes and co-transfected with the promoter luciferase reporter plasmid (450 ng) and pRL-TK (50 ng). At 48 h post-transfection, the cells were lysed and subjected to luciferase activity assays using the Dual-Glo System (Promega).

### Packaging of Virus Vectors

For delivering the reporter system into mice, we packaged lentiviruses through the triple-plasmid transfection method. HEK293T cells were co-transfected with the inserted promoter luciferase fragment plasmid pHAGE, envelope plasmid pMD2.G, and packaging plasmid psPAX. Then, the lentiviruses were harvested 48 h post-transfection, and we concentrated the lentivirus supernatants into a suitable volume (100 μl, over 1 × 10^8^ copies/ml) for injection according to the precipitation method using PEG-8000 (Sigma).

HBV-specific AAV8 delivery vector construction, viral packaging, and titration were performed by Beijing SyngenTech Co. Ltd. (Beijing, China). The efficient HBV gRNA T_2_ and gRNA T_6_ were separately cloned into the reconstructed vector pX601-EnhII-Pa1AT-SaCas9. Verification and sequencing confirmation of the plasmids were conducted by SyngenTech.

### Design and Cloning of the Liver-Specific and HBV-Targeting SaCas9 System

We designed seven functional gRNA sequences T_1_–T_7_, and with the exception of T_4_ and T_7_, the other gRNA sequences differed from similar publications (Liu et al., 2015; Li et al., 2018b). All sequences were derived from the conserved region of the HBV genome among different genotypes from A to H, which included the initiating 5'G and the downstream 3'PAM NGGRRT (GN20-NNGRRT). We separately replaced the original CMV promoter with four liver-specific promoters EnhI/X, PEPCK, EnhII-PEPCK, and EnhII-Pa1AT to drive cas9 expression in plasmid pX601.

### Detection of HBsAg and HBeAg

At the indicated time points, cell culture supernatants or mice sera were collected to detect the levels of HBsAg and HBeAg using a commercial ELISA kit (Kehua Bioengineering). All values were normalized against β-galactosidase activities in the cell lysates as measured using the Beta-Glo System (Promega).

### Quantitative RT-PCR

Hieff® qPCR SYBR Green Master Mix (Low Rox Plus) was used in quantitative PCR (qPCR). The primers used in this study are provided in Supplementary Table S1. For the quantification of HBV RNA, GFP RNA, and SaCas9 RNA, total RNA was reverse transcribed into cDNA using random primers (PrimeScript RT kit; Takara), and 2 μl of the cDNA was used for qPCR assay.

### DNA and RNA Hybridization

The extraction and analysis of HBV DNA and RNA were performed as previously described (Hao et al., 2015; Liu et al., 2015). Probe preparation and subsequent DIG detection were conducted using the DIG Northern Starter Kit (Roche Diagnostics, Indianapolis, IN, United States) according to the manufacturer’s instruction. The DIG-labeled plus strand-specific RNA probe corresponding to nucleotides 156–1,061 of the HBV genome was used for HBV DNA and RNA detection. 28S and 18S rRNA were used as loading controls.

### Statistical Analyses

All experiments were repeated at least three times. The results are presented as means ± SEM. The statistical significance differences were determined by using one-way ANOVA analysis with multiple comparison test and independent Student’s *t*-test. Statistical analyses were achieved using the Prism 8 software (GraphPad Software Inc., San Diego, CA, United States). A *p* < 0.05 was considered statistically significant.

## Results

### Design and Cloning of Liver-Specific and HBV-Targeting SaCas9 System

**Figure 1:**
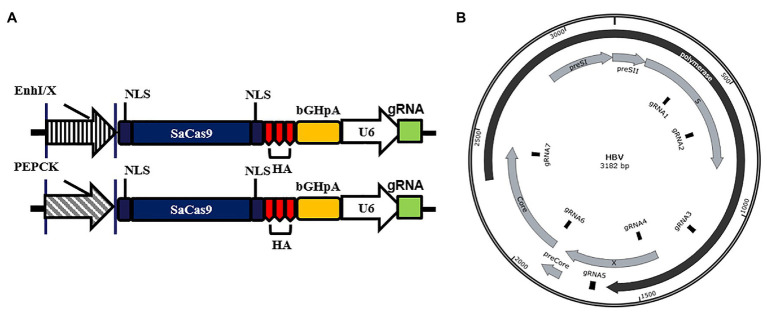
Design of liver-specific and hepatitis B virus (HBV)-targeting CRISPR/SaCas9 system. **(A)** Design of the reconstructed guide RNA (gRNA)/SaCas9-expressing vector pX601-U6-HBVgRNA plasmids. NLS, nuclear localization signal; HA, HA-tag; SaCas9, human codon-optimized *Staphylococcus aureus* Cas9; bGHpA, bovine growth hormone poly(A) signal. **(B)** Schematic diagram of the gRNA-targeted sequences located in the HBV genome.

To ensure that the gRNAs could target different HBV genotypes and reduce missing targets caused by viral genome mutations, we aligned the sequences of 22 representative HBV genotypes from the NCBI Viral Genomes Resource (Supplementary Figure S1). EnhI/X and PEPCK were selected to replace the CMV promoter in the gRNA/SaCas9-expressing vector pX601, as shown in Figure 1A. Based on the screening criteria mentioned in the Materials and Methods section, seven gRNAs targeting different regions of HBV genome were designed (Figure 1B; Table 1).

**Table 1:** Sequences of HBV-specific gRNAs.

Name	Sequence 5'-3'	Location
T_1_(gRNA1)	caccGTCCAACTTGTCCTGGTTATCG	354–374
T_2_(gRNA2)	caccGGGCTTTCGGAAAATTCCTAT	623–643
T_3_(gRNA3)	(−)caccGACCTGGCCGTTGCCGGGCAA	1,153–1,173
T_4_(gRNA4)	caccGTTTGTTTACGTCCCGTCGGCG	1,422–1,442
T_5_(gRNA5)	(−)caccGCGTTGACATTGCAGAGAGTCC	1,670–1,690
T_6_(gRNA6)	caccGCATGGACATCGACCCTTATA	1,901–1,921
T_7_(gRNA7)	caccGTCGCAGAAGATCTCAATCTC	2,417–2,437
T_eGFP_	caccGAGCTGGACGGCGACGTAAA	

The capital letters are guide RNA sequences, and the lower case letters are sticky ends for cloning.

### *In vitro* Inhibition of HBV by the CRISPR/SaCas9 System Under Control of a Single Liver-Specific Promoter

**Figure 2:**
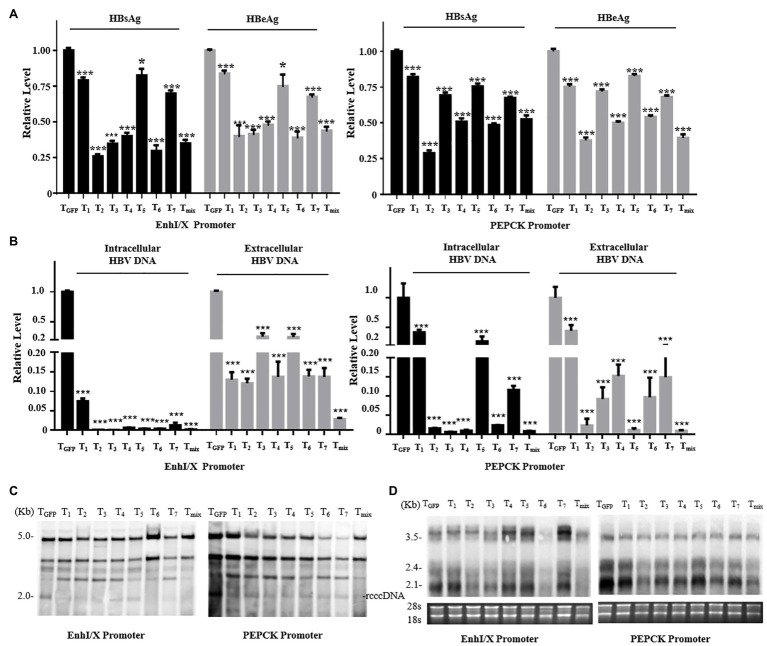
Anti-HBV effects of the reconstructed CRISPR/SaCas9 system under the control of single liver-specific promoter in Huh7 cells. Huh7 cells were co-transfected with reconstructed gRNA/SaCas9 system plasmids and reproduce rcccDNA system plasmids. **(A)** At 48 h post-transfection, the relative levels of HBsAg and HBeAg were measured by ELISA. **(B)** At 96 h post-transfection, intracellular HBV replication intermediates and extracellular virion DNA were extracted and the expression levels were measured using quantitative PCR (qPCR). **(C)** The expression levels of HBV intracellular replication were measured by Southern blotting. **(D)** The expression levels of the HBV transcripts were measured by Northern blotting. In each of the transfections, the pSV-β-gal plasmid was included to normalize the transfection efficiencies. The results of the ELISA and qPCR were calculated from three independent experiments, and the data are presented as the mean ± SEM. ^*^p < 0.05 and ^***^p < 0.001. T_mix_, the mixture of T_1_–T_7_.

To explore the anti-HBV effects of the reconstructed CRISPR/SaCas9 system with single liver-specific promoters, we selected promoters from both the virus and host to replace the CMV promoter. The vectors carrying T_1_–T_7_ or their mixture (T_mix_, seven gRNAs mixed in equal amounts) were co-transfected into Huh7 cells with the HBV genotype D to reproduce the rcccDNA system (prcccDNA-shB2M and pCMV-KRAB-Cre at a 1:1 ratio) and pSV-β-gal (as internal control). Comparison with the T_GFP_ (Target GFP gRNA) control group revealed that all gRNAs of two reconstructed CRISPR/SaCas9 systems reduced the average HBsAg and HBeAg levels in the supernatants by 25–85% (Figure 2A).

T_2_, T_3_, T_6_, and T_mix_ reduced the average HBsAg and HBeAg levels in the supernatant by more than one-half. Intracellular viral replication and extracellular offspring virion DNA were extracted and investigated using qPCR analysis. All gRNAs of the two reconstructed CRISPR/SaCas9 systems were found to dramatically suppress HBV replication (Figure 2B). Extracted DNA in the nucleus was detected by Southern blotting, and viral transcription was detected by Northern blotting. All gRNAs of the two reconstructed CRISPR/SaCas9 systems effectively reduced the amount of rcccDNA (similar to the natural cccDNA of HBV; Figure 2C), and the HBV RNA transcripts were stably reduced by T_2_, T_3_, and T_6_ (Figure 2D). These data suggest that the CRISPR/SaCas9 system containing a replacement liver-specific promoter still had evident *in vitro* inhibition of HBV, and T_2_, T_3_, and T_6_ were more effective than the other tested gRNAs.

### Study of the Tissue Tropism Effects of Candidate Liver-Specific Promoters *in vitro* and *in vivo*

**Figure 3:**
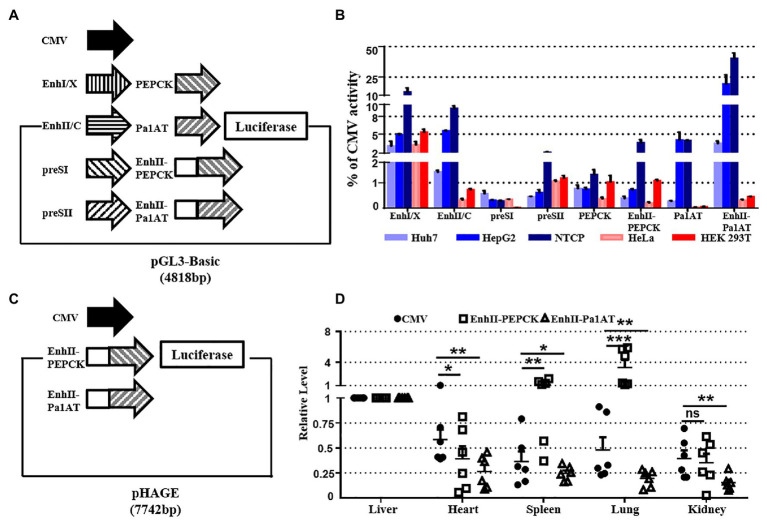
Activity of liver-specific promoters *in vitro* and *in vivo*. **(A)** Schematic of the reporter plasmids for studying specificity *in vitro*. Nine promoters were inserted into the pGL3-Basic plasmid. **(B)** Promoter activity in hepatoma and non-hepatocellular carcinoma cell lines. Hepatic (Huh7, HepG2, and NTCP) and non-hepatic (HeLa and HEK293T) cell lines were transiently transfected with reporter plasmids. **(C)** Schematic of the reporter plasmids for studying the specificity *in vivo*. Three luciferase linked promoters inserted into pHAGE plasmid for packaging lentivirus. **(D)** The mice were divided into three groups, and we injected the lentivirus-packaged luciferase reporter system. At 7 days post-injection, organ samples were harvested and luciferase mRNA was measured using RT-qPCR. The pRL-TK plasmid was included to normalize the transfection efficiencies, and Dual-Luciferase assay results were calculated from three independent experiments. The data are presented as the mean ± SEM. ^*^p < 0.05, ^**^p < 0.01, and ^***^p < 0.001, ns, no significant.

To screen for suitable liver-specific promoters, nine promoter fragments were inserted into pGL3-Basic (Figure 3A). EnhI/X (nt 950–1,375), EnhII/C (nt 1,415–1,815), preSI (nt 2,707–2,849), and preSII (nt 2,937–3,182) were from HBV (genotype D: GenBank accession no. V01460.1); PEPCK (540 bp) was from rat; and Pa1AT (305 bp) was derived from human. EnhancerII (EnhII, nt 1,621–1,775) was added at the 5' end of the host-derived promoter to increase the gene expression levels. CMV was used as a control promoter that can be widely and highly expressed in all cell types. To control the transfection efficiency, all the results in different cells were corrected for *Renilla* luciferase expression.

As negative controls, we used the human non-hepatocellular carcinoma cell lines HeLa and HEK293T. Of the four HBV promoters tested, EnhI/X was the strongest, yielding over 10% of CMV activity in NTCP; however, EnhII/C had the biggest difference in activity between hepatocellular carcinoma and non-hepatocellular carcinoma cell lines (Figure 3B). Overall, the expression of Pa1AT was higher than PEPCK, and the expression levels of both improved significantly after inclusion of EnhII. Among all the liver-specific promoters, EnhII-Pa1AT had the highest expression activity in hepatocellular carcinoma cell lines, showing the biggest difference expression between hepatoma and non-hepatocellular carcinoma cell lines.

To further test the *in vivo* specificity of the two liver-specific promoters derived from the host linked with a viral enhancer, we detected the expression of genes transduced using the lentiviral vector. Three promoters followed by luciferase were inserted into pHAGE (Figure 3C). RT-qPCR analysis was used to detect the luciferase mRNA driven by CMV and the other two chimeric promoters. Based on the expression levels in liver tissue, lower levels of expression were observed in heart, spleen, lung, and kidney with EnhII-Pa1AT compared to the CMV promoter (Figure 3D). However, the effect of EnhII-PEPCK was mediocre, even showing opposite liver specificity revealed by the results in spleen and lung. Taken together, these results demonstrate the higher activity and better specificity of the chimeric liver-specific promoter compared with the single liver-specific promoter. Especially, EnhII-Pa1AT demonstrated potential for further verification.

### Inhibition of HBV *in vitro* by the CRISPR/SaCas9 System Under the Control of Chimeric Liver-Specific Promoters

**Figure 4:**
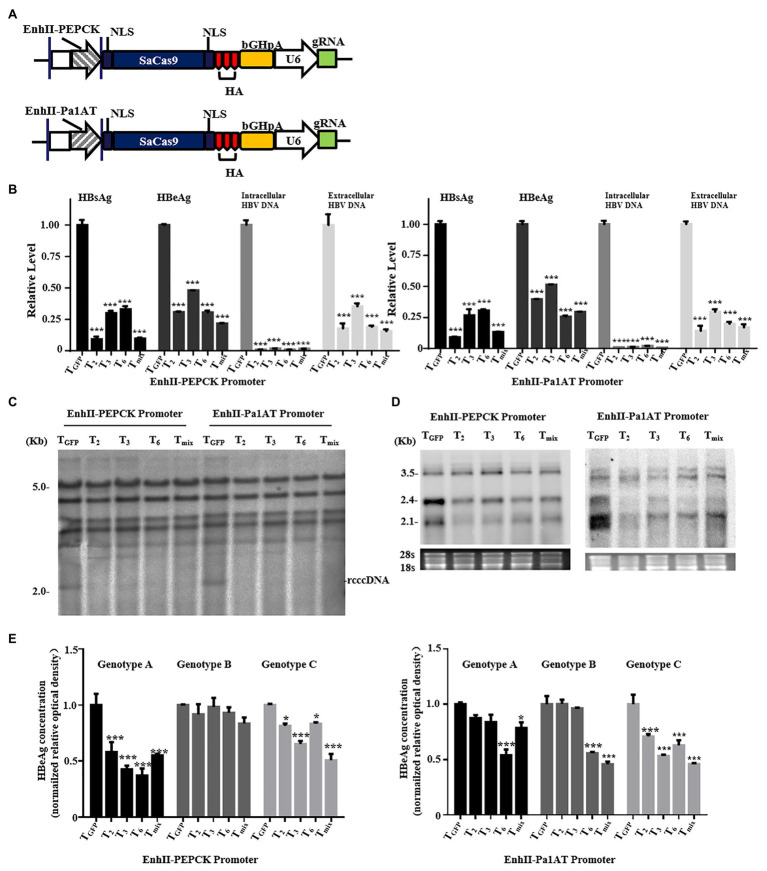
Anti-HBV effects of the CRISPR/SaCas9 system under the control of the chimeric liver-specific promoter in Huh7 cells. Huh7 cells were co-transfected with reconstructed gRNA/SaCas9 system plasmids and reproduce rcccDNA system plasmids. **(A)** Design of the reconstructed gRNA/SaCas9-expressing vector pX601-U6-HBVgRNA plasmids replaced with a chimeric liver-specific promoter. NLS, nuclear localization signal; HA, HA-tag; SaCas9, human codon-optimized *S. aureus* Cas9; bGHpA, bovine growth hormone poly(A) signal. **(B)** At 48 h post-transfection, HBsAg and HBeAg were measured using ELISA. The intracellular HBV replication intermediates and extracellular virion DNA were measured using qPCR at 96 h post-transfection. **(C)** At 48 h post-transfection, measurement of the HBV intracellular replication was through Southern blotting. **(D)** At 48 h post-transfection, measurement of the HBV RNA transcripts was by Northern blotting. **(E)** The genotype A, B, and C HBV replicons were co-transfected with the reconstructed gRNA/SaCas9 system plasmids. At 48 h post-transfection, the relative levels of HBeAg were measured using ELISA. In each of the transfections, the pSV-β-gal plasmid was included to normalize the transfection efficiencies. The results of ELISA and qPCR were calculated from three independent experiments and the data are presented as the mean ± SEM. ^*^p < 0.05 and ^***^p < 0.001. T_mix_, the mixture of T_2_, T_3,_ and T_6_.

To further explore the CRISPR/SaCas9 system reconstructed with chimeric liver-specific promoters, we replaced the CMV promoter of gRNA/SaCas9-expressing vector pX601 with EnhII-PEPCK and EnhII-Pa1AT (Figure 4A). For testing, we selected the three more-effective gRNAs (T_2_, T_3_, and T_6_) and their mixture (T_mix_ – the three gRNAs mixed in equal amounts) according to the previous results. Similar to the anti-HBV activity of the reconstructed CRISPR/SaCas9 system with the single liver-specific promoter, all parameters of the HBV lifecycle, including the HBsAg, HBeAg, HBV DNA, rcccDNA, and HBV RNA transcripts, were significantly repressed (Figures 4B–D).

To explore whether the target sequences showed broad-spectrum anti-HBV activity, we next verified the effects of the three selected gRNAs or T_mix_ for each reconstructed CRISPR/SaCas9 system using different HBV genotypes. The corresponding plasmids were co-transfected into Huh7 cells with each individual gRNA or mixture. The relative levels of HBeAg in the culture supernatants were measured using ELISA. The reconstructed CRISPR/SaCas9 system with EnhII-PEPCK effectively inhibited the HBeAg expression of HBV with genotypes A and C, and EnhII-Pa1AT could inhibit the HBeAg expression of HBV with genotype C to varying degrees (Figure 4E). However, T_6_ with EnhII-Pa1AT revealed an effective rate of almost 50% for the inhibition of HBV with the genotypes A, B, and C. According to this study of tissue tropism effects and the verification of anti-HBV *in vitro*, EnhII-Pa1AT was the most effective chimeric liver-specific promoter.

### Inhibition of HBV *in vivo* by the AAV8-Derived CRISPR/SaCas9 System With EnhII-Pa1AT Promoter

**Figure 5:**
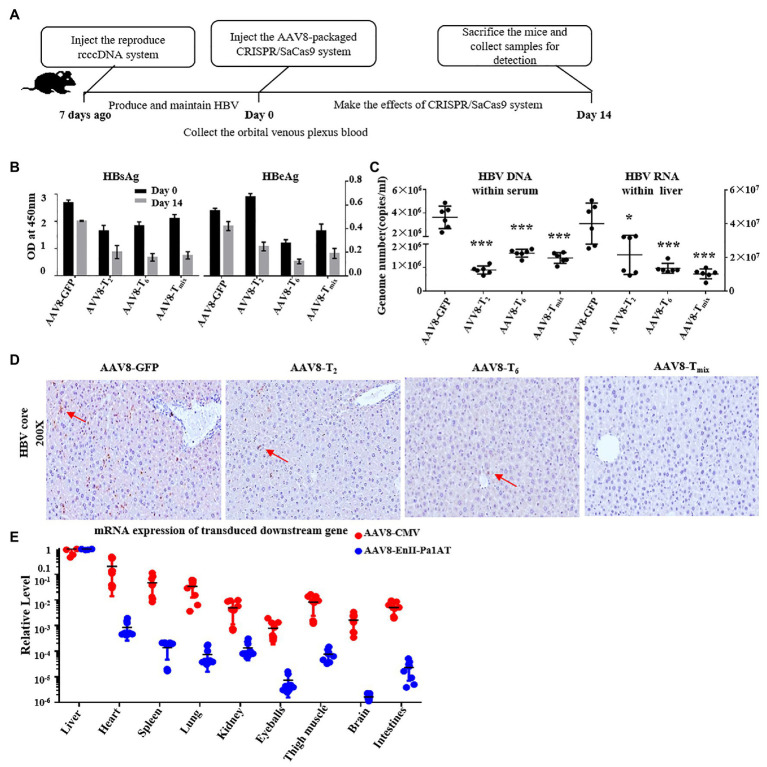
Anti-HBV effects of the CRISPR/SaCas9 system with the EnhII-Pa1AT promoter *in vivo*. **(A)** The mice were divided into four groups and subjected to hydrodynamic injection (HDI) with the reproduce rcccDNA system plasmids. At 7 days post-injection, we injected the adeno-associated virus (AAV)8-packaged CRISPR/SaCas9 system into the mice *via* the tail vein again. After two injections, blood samples were collected on day 0, and the mice were sacrificed on day 14. **(B)** The levels of HBsAg and HBeAg were measured using ELISA. **(C)** HBV DNA in the sera was measured using qPCR and HBV RNA in the liver was measured using RT-qPCR. **(D)** Immunohistochemical staining for HBcAg was performed in the liver. **(E)** Nine tissue samples were collected from twice injected mice as mentioned above, eGFP mRNA and SaCas9 RNA were measured using RT-qPCR. The results of the ELISA and qPCR were calculated from three independent experiments, and the data are presented as the mean ± SEM. ^*^p < 0.05 and ^***^p < 0.001. T_mix_, the mixture of AAV8-T_2_ and AAV8-T_6_.

To verify the inhibition efficiency of the reconstructed CRISPR/SaCas9 system with the EnhII-Pa1AT promoter *in vivo*, we used the C57BL/6 mice where HBV replication persists for a long time after injection with the reproduce rcccDNA system. We injected the reproduced rcccDNA system 1 week in advance for production and maintenance of HBV in mice through hydrodynamic injection (HDI), and then injected them again with AAV8 containing EnhII-Pa1AT-T_2_, EnhII-Pa1AT-T_6_, or EnhII-Pa1AT-T_mix_ (EnhII-Pa1AT-T_2_:EnhII-Pa1AT-T_6_ = 1:1) or AAV8 containing GFP. After 14 days, we harvested serum and liver samples from the mice (Figure 5A).

To eliminate the issue of differences in observations being due to differences in virus expression levels between groups of mice, we collected the orbital venous plexus blood before the second injection for detection of the HBsAg and HBeAg levels in the mice. Compared with the AAV8-delivered GFP expression control, the serum HBsAg levels fell even more with the AAV8-delivered CRISPR/SaCas9 system treatment, and AAV8-T_2_ had significant effects in inhibiting HBeAg (Figure 5B). HBV DNA in the serum and HBV RNA in liver were remarkably reduced after the administration of T_2_, T_6_, or T_mix_ (Figure 5C). As shown in Figure 5D, the expression of the HBV core protein antigen (brown color labeled by red arrow) in the mouse liver was also significantly inhibited by the AAV8-delivered CRISPR/SaCas9 system.

In the case of the reconstructed CRISPR/SaCas9 system delivered by the AAV8 vector, we collected the organs and tissues in addition to the liver (heart, spleen, lung, kidney, eyeball, thigh muscle, brain, and intestine). We analyzed the RNA levels of GFP and SaCas9 transgenes, which were driven by different promoters but delivered by the same tissue-specific AAV8 vector, using RT-qPCR. The expression of the target gene was significantly decreased in non-liver organs (Figure 5E). Under the action of the EnhII-Pa1AT promoter, the expression of the SaCas9 was further decreased in organs and tissues other than liver. In comparing the differences in transgene expression driven by the same promoter between the lentiviral vector and AAV8 vector, AAV8 had clear liver tropism effects.

## Discussion

We first investigated the anti-HBV effects of reconstructed CRISPR/SaCas9 in which the original promoter (CMV) was replaced with liver-specific promoters (Figures 2, 4, 5). Although the activity of liver-specific promoters for expression of the downstream gene was significantly weaker compared with the original CMV promoter (Figure 3B), the reconstructed CRISPR/SaCas9 still had significant anti-HBV suppression efficacy (Figures 2, 4). The use of multiple gRNAs (T_mix_) also demonstrated efficient inhibition of HBV. In fact, HBV polymerase lacks proofreading activity, i.e., does not have 3'-5' exonuclease activity. HBV is likely to mutate during reverse transcription, which increases its risk of escaping traditional antiviral drug treatment (Chan, 2011; Rajoriya et al., 2017). Therefore, more research is required on the use of multiple gRNAs to consider and discuss its potential ability to inhibit virus escape variants and the risks of off-target effects.

There have been numerous studies on liver-specific promoters. Daniel et al. quantitatively compared the *in vivo* levels of several liver-specific promoters, and their results indicated that hAAT had the strongest priming effect in reverse transcription vectors, which is of great significance for gene therapy (Hafenrichter et al., 1994b). As the effect of a single liver-specific promoter is much lower than that of the CMV promoter and enhancer, researchers often construct chimeric enhancers/promoters to regulate the transcription of a target gene.

Gabriela et al. linked the albumin enhancer (Ealb) and HBV enhancer (EII) to other promoters to construct different chimeric promoters (Kramer et al., 2003). Combining *in vitro* and *in vivo* data, studies have shown that Ealb-Pa1AT and EII-Pa1AT can continuously and efficiently induce gene expression in the liver and can be used as candidate promoters for gene therapy. Similar conclusions were obtained in our work, where EnhII-Pa1AT was observed to have the best efficiency and liver specificity at the cellular level among all the studied liver-specific promoters (Figure 3).

Compared to the CMV promoter, EnhII-Pa1AT had significantly reduced downstream gene expression in non-liver organs or tissues according to the results of the lentivirus and AAV vector transgene expression model. In addition, we found that the expression of downstream genes transduced by AAV vectors in non-liver organs was even less than the gene transduction by the lentivirus (Figures 3D, 5E). The replacement of liver-specific promoters or the use of hepatophilic AAV types can reduce the chance of cleavage in other organs or tissues, thereby reducing the possibility of pernicious targets as a whole.

Powerful models that can generate substantial cccDNA supercoils both *in vitro* and *in vivo* and with high efficiency and a long half-life are an important research requirement for HBV. Several recombinant cccDNA (rcccDNA) systems based on site-specific DNA recombination were developed (Guo et al., 2016; Li et al., 2016; Yan et al., 2017). These rcccDNAs were generated in large quantities and were heat stable and epigenetically organized as a mini-chromosome, with the unique attribute of establishing HBV persistence in immunocompetent mice. Such a system also represents a useful model for *in vitro* and *in vivo* evaluation of antiviral treatments against HBV cccDNA. We could easily determine the reduction of rcccDNA produced by the reproduced rcccDNA system through Southern blot in our study. In the mouse experiment, in contrast to operations in cell experiments and other hydrodynamically injected mice models, we injected a virus plasmid and CRISPR/SaCas9 1 week apart. This method without co-injection is more in line with a realistic virus treatment situation.

CRISPR variants have been continually identified and engineered for enhanced efficiency, decreased off-target editing, and reduced immunogenicity or size. These novel CRISPR variants, such as xCas9, Cas12a, and CasF, have great potential for gene therapy applications (Zetsche et al., 2016; Pausch et al., 2020; Zhang et al., 2020). Off-target detection still remains a key challenge, and this was also a limitation of this study. A variety of sequencing-based approaches are used to detect and quantify off-target effects caused by CRISPR (Manghwar et al., 2020). To comprehensively assess the risk of off-target effects, genome-wide sequencing is a popular choice (Klimke et al., 2019; Yu and Wu, 2019). Akcakaya et al. (2018) described verification of *in vivo* off-targets (VIVO), a highly sensitive strategy that can identify the genome-wide off-target effects of CRISPR/Cas nucleases *in vivo*. As the CRISPR system and methods for demonstrating off-target risks continue to be developed, its therapeutic potential will continue to increase.

## Data Availability Statement

The original contributions presented in the study are included in the article/Supplementary Material, further inquiries can be directed to the corresponding authors.

## Ethics Statement

The animal study was reviewed and approved by the Institutional Animal Care and Use Committee of Wuhan University (project license WDSKY0201802).

## Author Contributions

YC, KY, LmZ, and XL conceptualized the study design. KY, JF, JL, and TX collected the experiment result. HW and QL assisted in mouse experiment. KY plotted the figures, analyzed the data, and wrote the initial drafts of the manuscript. YC, KY, LiZ, and JF revised the manuscript and MS and HU commented on it. All authors contributed to the article and approved the submitted version.

### Conflict of Interest

The authors declare that the research was conducted in the absence of any commercial or financial relationships that could be construed as a potential conflict of interest.

## SupplementaRy Material

Supplementary Figure S1 Sequence alignment of HBV-specific gRNAs and their target sequences. The sequence of the HBV G genotype marked 36 bp was omitted.

Supplementary Table S1 Primers for qPCR.
