# Supplementary figures and images for: Inhibition of Hepatitis B Virus by AAV8-Derived CRISPR/SaCas9 Expressed From Liver-Specific Promoters
Source: Front Microbiol. 2021 Jun 26;12:665184. doi: 10.3389/fmicb.2021.665184 (PMC8271097; doi:10.3389/fmicb.2021.665184)

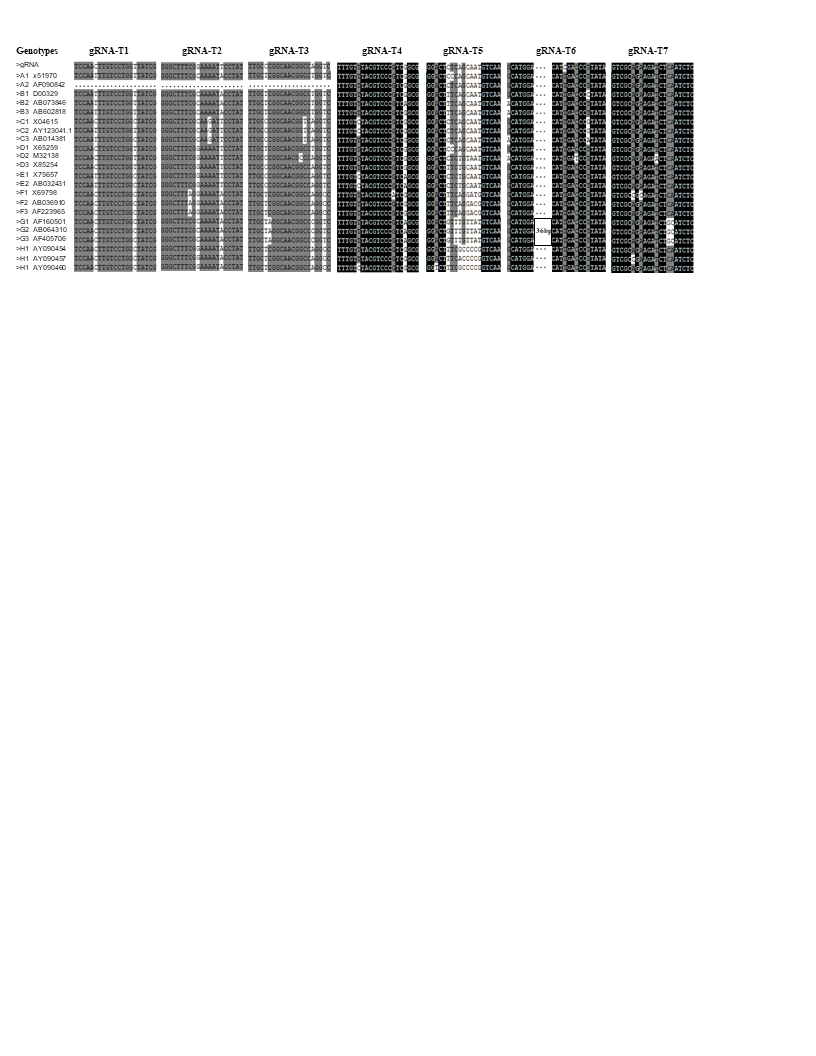

Supplement: Supplementary Figure S1 — Sequence alignment of HBV-specific gRNAs and their target sequences. The sequence of the HBV G genotype marked 36 bp was omitted. [file Image_1.TIF]

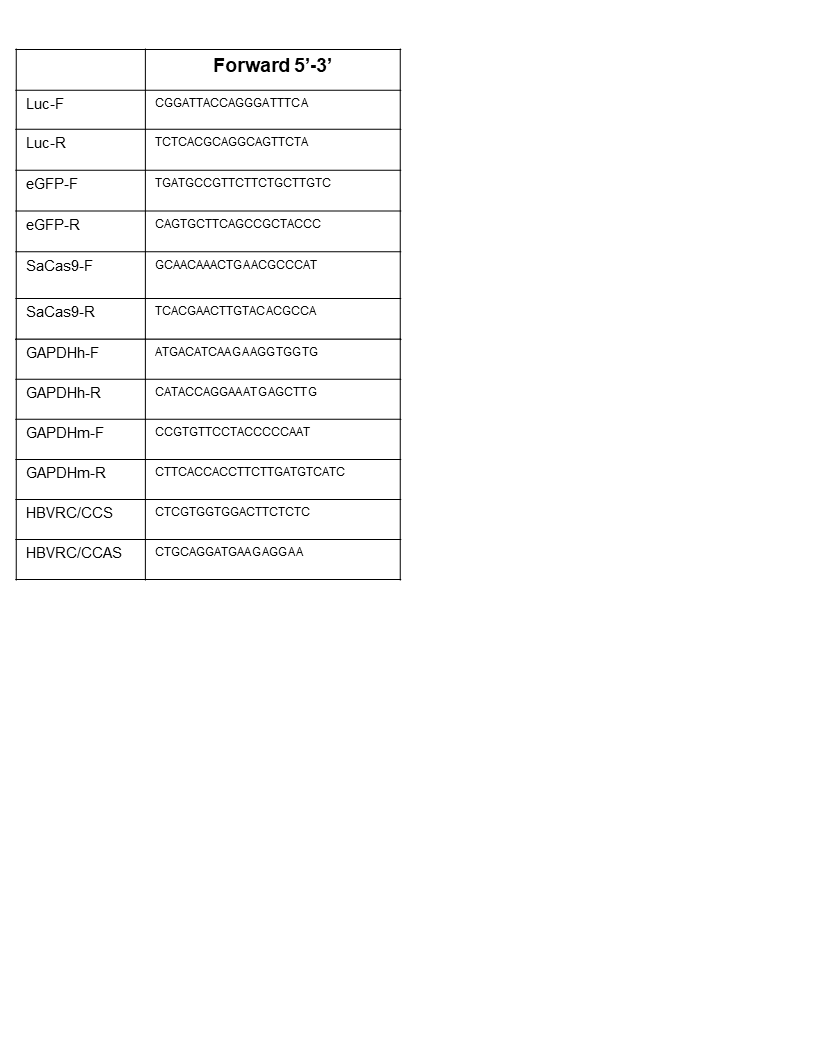

Supplement: Supplementary Table S1 — Primers for qPCR. [file Image_2.TIF]
